# Modeling light response of effective quantum efficiency of photosystem II for C_3_ and C_4_ crops

**DOI:** 10.3389/fpls.2025.1478346

**Published:** 2025-03-06

**Authors:** Xiao-Long Yang, Ting An, Zi-Wu-Yin Ye, Hua-Jing Kang, Piotr Robakowski, Zi-Piao Ye, Fu-Biao Wang, Shuang-Xi Zhou

**Affiliations:** ^1^ School of Life Sciences, Nantong University, Nantong, China; ^2^ State Key Laboratory of Environmental Chemistry and Ecotoxicology, Research Center for Eco-Environmental Sciences, Chinese Academy of Sciences, Beijing, China; ^3^ College of Bioscience and Bioengineering, Jiangxi Agricultural University, Nanchang, China; ^4^ School of Foreign Languages, Guangdong Baiyun University, Guangzhou, China; ^5^ Key Laboratory of Crop Breeding in South Zhejiang, Wenzhou Academy of Agricultural Sciences, Wenzhou, China; ^6^ Department of Forestry and Wood Technology, Poznan University of Life Sciences, Poznan, Poland; ^7^ New Quality Productivity Research Center, Guangdong ATV College of Performing Arts, Deqing, China; ^8^ Math & Physics College, Jinggangshan University, Ji’an, China; ^9^ Department of Biological Sciences, Macquarie University, Sydney, NSW, Australia

**Keywords:** Ye model, effective quantum efficiency of photosystem II (Φ_PSII_), non-photochemical quenching, light absorption cross-section, light-harvesting pigment molecules, photosynthetic light response

## Abstract

Effective quantum efficiency of photosystem II (Φ_PSII_) represents the proportion of photons of incident light that are actually used for photochemical processes, which is a key determinant of crop photosynthetic efficiency and productivity. A robust model that can accurately reproduce the nonlinear light response of Φ_PSII_ (Φ_PSII_–*I*) over the I range from zero to high irradiance levels is lacking. In this study, we tested a Φ_PSII_–*I* model based on the fundamental properties of light absorption and transfer of energy to the reaction centers via photosynthetic pigment molecules. Using a modeling-observation intercomparison approach, the performance of our model versus three widely used empirical Φ_PSII_–*I* models were compared against observations for two C_3_ crops (peanut and cotton) and two cultivars of a C_4_ crop (sweet sorghum). The results highlighted the significance of our model in (1) its accurate and simultaneous reproduction of light response of both Φ_PSII_ and the photosynthetic electron transport rate (*ETR*) over a wide I range from light limited to photoinhibition *I* levels and (2) accurately returning key parameters defining the light response curves.

## Introduction

Light intensity (*I*; see **Table 1** for list of abbreviations and definitions) exhibits dynamic fluctuations across various temporal scales, ranging from seconds to months due to wind-induced leaf movements and diurnal solar variations (Liu et al., 2021). Under low *I* conditions, plants efficiently channel the majority of absorbed light to reaction centers for photochemical processes. Under high *I* conditions, plants dissipate approximately 80% of absorbed light as heat through non-photochemical quenching (*NPQ*) to prevent light damage (Niyogi and Truong, 2013). The generation of *NPQ* primarily originates from the de-excitation of light-harvesting pigment molecules in excited state (*N*_k_). Consequently, there exists a significant correlation between the quantity of *N*_k_ and the magnitude of *NPQ*. Additionally, the light environment within leaves is highly heterogeneous due to the focusing effect of epidermal cells and the light-guiding properties of vascular bundle sheath structures (Xiao et al., 2016; Song et al., 2017). This heterogeneity results in significant *I* variations among different cells within the leaf and even among chloroplasts within the same mesophyll cell (Xiao et al., 2016). Photosystems within chloroplasts constantly operate in a highly dynamic light environment, and accurate modeling of photosynthetic responses to rapid change of *I* is important for us to understand the adaptive responses of plants to the changing light environments.

**Table 1:** List of major model parameters defining the light response curves of effective quantum efficiency of photosystem II (Φ_PSII_) and electron transport rate (*ETR*).

Symbol	Definition	Unit
*ETR*	Electron transport rate	μmol electrons m^−2^ s^−1^
*ETR*_max_	Maximum electron transport rate	μmol electrons m^−2^ s^−1^
*F*	Steady-state fluorescence	
*F*_m_	Maximum fluorescence in the dark adaptation	
*F*_m_'	Maximum fluorescence in the light	
*F*_v_	Variable fluorescence yield of the dark-adapted leaf PSII	
*F*_v_/*F*_m_	Maximum quantum efficiency of PSII	
*g*_i_	Degeneration of energy level of photosynthetic pigment molecules in the ground state *i*	
*g*_k_	Degeneration of energy level of photosynthetic pigment molecules in the excited state *k*	
*I*	Photon flux densities or light intensity	μmol photons m^−2^ s^−1^
*k*_P_	Rate of photochemical reaction	s^−1^
*k*_D_	Rate of heat loss	s^−1^
*α*_e_	Initial slope of the light response curve of electron transport rate	μmol electrons (μmol photons)^−1^
*β*_e_	Dynamic down-regulation term of PSII	m^2^ s (μmol photons)^−1^
*γ*_e_	Saturation term of photosynthesis	m^2^ s (μmol photons)^−1^
*NPQ*	Non-photochemical quenching	
*a*	Initial slope of the light response curve of non-photochemical quenching	
*b*	*b* is equivalent to *γ*_e_, the saturation term of photosynthesis	
*NPQ*_0_	Total light-harvesting pigment molecules	
*N*_k_	Total light-harvesting pigment molecules in excited state *k*	
*I*_sat_	Saturation light intensity corresponding to *ETR*_max_	μmol photons m^−2^ s^−1^
*ξ*_1_	Probability of photochemistry	
*ξ*_2_	Probability of heat loss	
*ξ*_3_	Probability of fluorescence	
*R*_ki_	Relaxation rate by spontaneous emission from excited state *k* to ground state *i*	s^−1^
*σ*_ik_	Eigen-absorption cross-section of photosynthetic pigment from ground state *i* to excited state *k* due to light illumination	m^2^
*σ*_'ik_	Effective absorption cross-section of light-harvesting pigment molecules	m^2^
*φ*	Exciton-use efficiency in PSII	
*τ*	Average lifetime of the photosynthetic pigment molecules in the lowest excited state	s
Φ_PSII_	Effective quantum efficiency of PSII	
Φ_PSIImax_	Maximum effective quantum efficiency of PSII	

Photosystem II (PSII) is pivotal in the light-dependent reactions of photosynthesis, driving the initial steps of energy conversion. Its activity can be conveniently assayed using bio-optical techniques (Buckley and Farquhar, 2004; Robakowski, 2005; Baker, 2008; Pavlovič et al., 2011; Shevela et al., 2023), with chlorophyll *a* fluorescence being the most widely adopted method. This technique facilitates the research into several key photosynthetic properties, including the maximum quantum efficiency of PSII (*F*_v_/*F*_m_), the effective quantum efficiency of PSII [Φ_PSII_ = (*F*_m_′ − *F*′)/*F*_m_′, where *F*_m_′ is the maximum fluorescence in the light and *F*′ is steady-state fluorescence], the photosynthetic electron transport rate (*ETR*) and *NPQ*. In addition, given that the generation of *NPQ* mainly results from the de-excitation of *N*_k_, the concurrent change in *N*_k_ and *NPQ* with the increasing *I* should be able to be characterized with a robust model.

Φ_PSII_ and *ETR* are the most widely used photochemical parameters to assess the efficiency of plant photochemistry in different environments (Genty et al., 1989; Moin et al., 2016). Φ_PSII_ represents the proportion of photons of incident light that are actually used to drive photochemistry, and it is closely linked with the closure and opening of PSII in photosynthetic primary reactions and chlorophyll fluorescence emission (Baker, 2008). Meanwhile, *ETR* is closely related to Φ_PSII_ and *I* (Genty et al., 1989; Krall and Edwards, 1992). Many studies using fluorescence techniques to determine Φ_PSII_ found it decreasing nonlinearly with the increasing *I* (Robakowski, 2005; Pavlovič et al., 2011; van der Tol et al., 2014; Córdoba et al., 2016). With the increasing *I*, *ETR* initially increased, and then, it reached a platform or there occurred photoinhibition or dynamic downregulation of PSII at high light intensities. This nonlinear relationship reflects the complex interplay between light absorption, energy transfer, and dissipation mechanisms in the photosynthetic apparatus, highlighting the adaptive responses of plants to varying environments.

No model has yet been reported to simultaneously accurately reproduce Φ_PSII_ *–I* and *ETR–I* curves over a wide I range from zero to photoinhibitory *I* levels. Among the limited studies characterizing the Φ_PSII_ *–I* curve, the negative exponential function and the exponential function are the most widely used models (Webb et al., 1974; Smyth et al., 2004; Ritchie, 2008; Ritchie and Bunthawin, 2010; Silsbe and Kromkamp, 2012; Robakowski et al., 2018). However, it has been reported that the values of *I*_sat_ estimated by the negative exponential functions are significantly higher than the measured values (Robakowski, 2005; Ritchie, 2008; Ritchie and Bunthawin, 2010). In addition, the non-rectangular hyperbolic model (NRH model) is the most widely used to characterize the *ETR–I* curve and returning *ETR*_max_ (von Caemmerer, 2000; Long and Bernacchi, 2003; Miao et al., 2009; Yin et al., 2009; Gu et al., 2010; Bernacchi et al., 2013; von Caemmerer, 2013; Buckley and Diaz-Espejo, 2015; Cai et al., 2018; Yin et al., 2021). However, the NRH model significantly have been reported to overestimate *ETR*_max_, and it cannot return a realistic *I*_sat_ due to its asymptotic function (Buckley and Diaz-Espejo, 2015; Yang et al., 2024). Experimentally, the value of Φ_PSIImax_ was measured when *I* was 0 μmol photons m^−2^ s^−1^.

In this study, we aimed to develop and test a Φ_PSII_–*I* model based on the fundamental properties of light absorption of photosynthetic pigment molecules (Ye et al., 2013a, 2013b). We evaluated the performance of the model using a modeling-observation intercomparison approach against observations conducted on two C_3_ corps (peanut and cotton) and two genotypes of a C_4_ crop (sweet sorghum cultivars KFJT-1 and KFJT-4). We also compared the robustness of this model against three widely used empirical *ETR–I* and Φ_PSII_–*I* models (i.e., the negative exponential function, the exponential function, and the NRH model) in their performances of (1) reproducing the observed light response curves and (2) returning key parameters defining the curves (i.e., Φ_PSIImax_, *ETR*_max_ and the corresponding *I*_sat_).

## Materials and methods

### Plant materials

Seeds of peanut (*Arachis hypogaea* L.) and cotton (*Gossypium hirsutum* L.) were surface disinfected with 70% ethanol and 20% bleach, then planted in trays and placed in an RDN-1000E-4 growth chamber (Ningbo Dongnan Instrument Co., China) under conditions of 23°C and 28°C (16 h/8 h light/dark cycle) for cultivation. When the seedlings developed two cotyledons, they were transplanted into the fields of the Botanical Garden at Nantong University. Field management was carried out according to the previously described methods (Wang et al., 2017). Seedling cultivation of sweet sorghum (*Sorghum bicolor* L. Moench, KFJT-1 and KFJT-4) followed the protocols established in our previous research (Yang et al., 2024). These two strains were developed by the Institute of Modern Physics, Chinese Academy of Sciences, through heavy ion irradiation of the parental line KFJT-CK. The cultivated seedlings were transferred to plastic pots and placed in a climate-controlled chamber, where they were grown under 25,000 lx light intensity, at 25°C, and with a 16 h/8 h light/dark cycle. Healthy plants bearing eight leaves were selected for data measurements for each species.

### Analytical models

#### Model 1 (Ye model)

The Ye model provides a mechanistic framework for describing the light response of *ETR* in PSII based on the biophysical properties of light-harvesting pigment molecules using Equation 1 (Ye et al., 2013a, 2013b):


(1) $$ETR=\frac{\alpha \beta N_{0}\sigma_{\text{ik}}\varphi }{S}\times \frac{1-\frac{(1-\frac{g_{i}}{g_{k}})\sigma_{\text{ik}}\tau }{\xi_{3}+(\xi_{1}k_{\text{P}}+\xi_{2}k_{\text{D}})\tau }I}{1+\frac{(1-\frac{g_{i}}{g_{k}})\sigma_{\text{ik}}\tau }{\xi_{3}+(\xi_{1}k_{\text{P}}+\xi_{2}k_{\text{D}})\tau }I}I$$

where *φ* is the exciton-use efficiency in PSII, *N*_0_ is total photosynthetic pigment molecules of the measured leaf, *S* is the leaf area (m^2^), and *g*_i_ and *g*_k_ are the degeneration of energy levels of photosynthetic pigments in the ground state (*i*) and excited state (*k*), respectively. *k*_P_ and *k*_D_ are rates of the photochemical reaction and heat loss, respectively (Baker, 2008). *ξ*_1_, *ξ*_2_, and *ξ*_3_ are the occupation probability of photochemistry, heat loss, and fluorescence emission, respectively. *σ*_ik_ is the eigen-absorption cross-section of photosynthetic pigments from the ground state *i* to the excited state *k* via light exposure, and *τ* is the average lifetime of the photosynthetic pigments in the lowest excited state *k*.

To simplify Equation 1, three aggregate parameters encapsulating biophysical dynamics are introduced: $\alpha_{\text{e}}=\frac{\alpha \beta N_{0}\sigma_{\text{ik}}\varphi }{S}$ [μmol electrons (μmol photons)^−1^] represents the initial slope of the light response curve of electron transport rate (*ETR–I* curve), $\beta_{\text{e}}=\frac{(1-\frac{g_{\text{i}}}{g_{\text{k}}})\sigma_{\text{ik}}\tau }{\xi_{3}+(\xi_{1}k_{\text{P}}+\xi_{2}k_{\text{D}})\tau }$ [m^2^ s (μmol photons)^−1^] reflects the dynamic downregulation term of PSII, and $\gamma_{\text{e}}=\frac{(1+\frac{g_{\text{i}}}{g_{\text{k}}})\sigma_{\text{ik}}\tau }{\xi_{3}+(\xi_{1}k_{\text{P}}+\xi_{2}k_{\text{D}})\tau }$ [m^2^ s (μmol photons)^−1^] represents the saturation term of photosynthesis.

With these parameters, Equation 1 simplifies to:


(2) $$ETR=\alpha_{\text{e}}\frac{1-\beta_{\text{e}}I}{1+\gamma_{\text{e}}I}I$$

Equations 1 and 2 describe *ETR–I* function and depict the interdependence between *ETR* and biophysical parameters.

Since Equation 1 is a non-asymptotic function, it has the first derivative. When the first derivative of Equation 1 equals to zero, *I*_sat_ is calculated as follows:


(3) $$I_{\text{sat}}=\frac{\sqrt{\frac{\left(\beta_{\text{e}}+\gamma_{\text{e}}\right)}{\beta_{\text{e}}}}-1}{\gamma_{\text{e}}}$$

Substituting Equation 3 into Equation 2, the maximum *ETR* (*ETR*_max_) can be determined using Equation 4:


(4) $$ETR_{\text{max}}=\alpha_{\text{e}}{\left(\frac{\sqrt{\beta_{\text{e}}+\gamma_{\text{e}}}-\sqrt{\beta_{\text{e}}}}{\gamma_{\text{e}}}\right)}^{2}$$

Moreover, combing Equation 1 with *ETR* =*α*×*β*×Φ_PSII_×*I* (Krall and Edwards, 1992), the relationship between Φ_PSII_ and *I* can be described as follows:


(5) $$\Phi_{\text{PSII}}=\frac{N_{0}\sigma_{\text{ik}}\varphi }{S}\times \frac{1-\frac{(1-\frac{g_{i}}{g_{k}})\sigma_{\text{ik}}\tau }{\xi_{3}+(\xi_{1}k_{\text{P}}+\xi_{2}k_{\text{D}})\tau }I}{1+\frac{(1-\frac{g_{i}}{g_{k}})\sigma_{\text{ik}}\tau }{\xi_{3}+(\xi_{1}k_{\text{P}}+\xi_{2}k_{\text{D}})\tau }I}I$$

Simplified, this becomes:


(6) $${\text{Φ}}_{\text{PSII}}={\text{Φ}}_{\text{PSIImax}}\frac{1-\beta_{\text{e}}I}{1+\gamma_{\text{e}}I}$$

where ${\text{Φ}}_{\text{PSIImax}}=\frac{\alpha_{\text{e}}}{\alpha \beta }$ .

The effective absorption cross-section of light-harvesting pigment molecules (*σ′*_ik_), which represents its ability to absorb light energy with *I*, can also be expressed as a function of *I* (Ye et al., 2013b). Namely,


(7) $${\text{σ}}_{\text{ik}}^{'}=\frac{\sigma_{\text{ik}}}{1+\frac{(1-\frac{g_{\text{i}}}{g_{\text{k}}})\sigma_{\text{ik}}\tau I}{\xi_{3}+(\xi_{1}k_{\text{P}}+\xi_{2}k_{\text{D}})\tau }}\;\times [1-\frac{(1-\frac{g_{\text{i}}}{g_{\text{k}}})\sigma_{\text{ik}}\tau I}{\xi_{3}+(\xi_{1}k_{\text{P}}+\xi_{2}k_{\text{D}})\tau }]$$

Equation 7 shows that *σ′*_ik_ increases with *k*_P_, *k*_D_, *ξ*_1_, *ξ*_2_, *ξ*_3_, and 1/*τ* but decreases with *I*. *σ′*_ik_ = *σ*_ik_ when *I* = 0 μmol photons m^−2^ s^−1^. As such, the light absorption cross-section is not a constant under any given *I* (excluding *I* = 0 μmol photons m^−2^ s^−1^). By introducing *β*_e_ and *γ*_e_, Equation 7 can be simplified to:


(8) $${\text{σ}}_{\text{ik}}^{'}=\frac{1-\beta_{\text{e}}I}{1+\gamma_{\text{e}}I}\sigma_{\text{ik}}$$

Comparing Equation 5 with Equation 7, the relationship between Φ_PSII_ and *σ′*_ik_ is described by Equation 9:


(9) $${\text{Φ}}_{\text{PSII}}={\text{Φ}}_{\text{PSIImax}}\frac{{\text{σ}}_{\text{ik}}^{'}}{\sigma_{\text{ik}}}$$

For a given species under given environmental conditions, the values of Φ_PSIImax_ and *σ*_ik_ are constants. Equation 9 demonstrates that Φ_PSII_ is directly proportional to *σ′*_ik_, and it changes as a function of *σ′*_ik_.

The number of photosynthetic pigment molecules in the excited state *k* (*N*_k_) can be expressed as:


(10) $$N_{\text{k}}=\frac{\sigma_{ik}\tau I}{\left(1-\frac{g_{\text{i}}}{g_{\text{k}}})\sigma_{\text{ik}}\tau I+(\xi_{3}+\xi_{1}k_{\text{P}}\tau +\xi_{2}k_{\text{D}}\tau \right)}N_{0}$$

Equation 10 demonstrates that *N*_k_ is a dynamic variable, exhibiting continuous fluctuations rather than maintaining constant values. *N*_k_ decreases with *k*_P_, *k*_D_, *ξ*_1_, *ξ*_2_, and *ξ*_3_ but increases with *σ*_ik_, *τ*, and *I*. Under dark conditions (*I* = 0 μmol photons m^−2^ s^−1^), it must be that *N*_k_ equals 0.

By applying *β*_e_ and *γ*_e_ to Equation 10, it can be simplified to:


(11) $$N_{\text{k}}=\;\frac{1}{1-\frac{g_{\text{i}}}{g_{\text{k}}}}\times \frac{\beta_{\text{e}}I}{1+\gamma_{\text{e}}I}N_{0}$$

Equation 11 shows that *N*_k_ increases with the increasing *I*.

Considering that chlorophyll fluorescence primarily stems from light-harvesting pigment molecules in the excited state, it is logical to infer that *NPQ* response to *I* would closely mirror the response of *N*_k_ to *I*. Building on Equation 11, the light response expression for *NPQ* can be deduced from the light response model of *N*_k_, the expression for *NPQ* in response to light can be derived as:


(12) $$NPQ=NPQ_{\text{max}}\frac{aI}{1+bI}+NPQ_{0}$$

where $a=\frac{\beta_{\text{e}}}{1-\frac{g_{\text{i}}}{g_{\text{k}}}}$ is initial slope of the light response curve of *NPQ*, $b=\frac{(1-\frac{g_{\text{i}}}{g_{\text{k}}})\sigma_{\text{ik}}\tau }{\xi_{3}+(\xi_{1}k_{\text{P}}+\xi_{2}k_{\text{D}})\tau }$ is equivalent to *γ*_e_, the saturation term of photosynthesis, and *NPQ*_0_ is *NPQ* at *I* = 0 μmol photons m^−2^ s^−1^.

Equations 2, 5 (and 6), 8, 11, and 12 represent how Model 1 (Ye model) describes Φ_PSII_–*I*, *ETR*–*I*, *σ′*_ik_–*I N*_k_–*I*, and *NPQ*–*I* curves, respectively.

#### Model 2 (negative exponential function)

It has been found experimentally that Φ_PSII_, ranging from 0 to 1, usually follows a simple negative exponential function as follows (Ritchie, 2008; Ritchie and Bunthawin, 2010; Buckley and Diaz-Espejo, 2015):


(13) $${\text{Φ}}_{\text{PSII}}={\text{Φ}}_{\text{PSIImax}}\times e^{-k_{\text{w}}I}$$

where Φ_PSIImax_ is defined as the maximum effective quantum efficiency when *I* = 0 μmol photons m^−2^ s^−1^, *k*_w_ is a scaling constant, and *I* is the light intensity. The values of Φ_PSIImax_ and *k*_w_ can be obtained when Φ_PSII_–*I* curves are simulated by Equation 13.

Substituting Equation 13 into *ETR* = *α*×*β*×Φ_PSII_×*I* (Krall and Edwards, 1992), we get the following expression for *ETR*:


(14) $$ETR=\alpha \times \beta \times I\times {\text{Φ}}_{\text{PSIImax}}\times e^{-k_{\text{w}}I}$$

When the first derivative of Equation 14 equals to zero, we can calculate saturation *I* (*I*_sat_=1/*k*_w_), then substitute *k*_w_ =1/*I*_sat_ into Equation 14 to determine the maximum electron transport rate (*ETR*_max_ = *α*×*β*×*I*_sat_×Φ_PSIImax_×e^−1^).

Equations 13 and 14 represent how the negative exponential function (Model 2) describes Φ_PSII_–*I* and *ETR*–*I* curves. It should be noted that there are two values of Φ_PSIImax_ to be returned when both Φ_PSII_–*I* and *ETR*–*I* curves are simulated by Equations 13 and 14, respectively.

#### Model 3 (exponential function)

The exponential function was introduced to simulate Φ_PSII_–*I* curves as follows (Smyth et al., 2004; Silsbe and Kromkamp, 2012):


(15) $${\text{Φ}}_{\text{PSII}}=\frac{F_{\text{v}}}{F_{\text{m}}}\times \frac{I_{\text{sat}}}{I}[1-\text{exp}(-\frac{I}{I_{\text{sat}}})]$$

where *F*_v_/*F*_m_ is the “dark-adapted” maximum operating efficiency of PSII, and *I*_sat_ is the saturation *I* (Smyth et al., 2004). However, it should be noted that Φ_PSIImax_ cannot be estimated by Equation 15, but *I*_sat_ and *F*_v_/*F*_m_ can be estimated when Φ_PSII_–*I* curve is fitted by Equation 15.

Similarly, substituting Equation 15 into *ETR* = *α*×*β*×Φ_PSII_×*I* (Krall and Edwards, 1992), we get the following expression for *ETR*:


(16) $$ETR=\alpha \times \beta \times \frac{F_{\text{v}}}{F_{\text{m}}}\times I_{\text{sat}}[1-\text{exp}(-\frac{I}{I_{\text{sat}}})]$$

The values of *I*_sat_ and *F*_v_/*F*_m_ can be returned when *ETR*–*I* curves are simulated by Equation 16.

The maximum *ETR* can be calculated by Equation 17:


(17) $$ETR_{\text{max}}=\alpha \times \beta \times \frac{F_{\text{v}}}{F_{\text{m}}}\times I_{\text{sat}}[1-\text{exp}(-1)]$$

Equations 15 and 16 represent how the exponential function (Model 3) describes Φ_PSII_–*I* and *ETR*–*I* curves. Similarly, it should be noted that there are two values of *I*_sat_ and *F*_v_/*F*_m_ to be returned when both Φ_PSII_–*I* and *ETR*–*I* curves are simulated by Equations 15 and 16, respectively.

#### Model 4 (non-rectangular hyperbolic model)

The non-rectangular hyperbolic (NRH) model has been mainly used to fit the *ETR*–*I* curves of plants (von Caemmerer, 2000; Long and Bernacchi, 2003; Miao et al., 2009; Yin et al., 2009; Gu et al., 2010; Bernacchi et al., 2013; von Caemmerer, 2013; Buckley and Diaz-Espejo, 2015; Cai et al., 2018; Yin et al., 2021), and it has been a sub-model in the FvCB model when irradiance is below the saturation level (Farquhar et al., 1980; von Caemmerer, 2000, 2013; Park et al., 2016; Yin et al., 2021). In the NRH model, the dependence of *ETR* on *I* can be expressed as follows:


(18) $$ETR=\frac{\alpha^{\prime }\times I+ETR_{\text{max}}-\sqrt{{\left(\alpha^{\prime }\times I+ETR_{\text{max}}\right)}^{2}-4\theta \times \alpha^{\prime }\times I\times ETR_{\text{max}}}}{2\theta }$$

where *α′* is defined as the initial slope of the *ETR–I* curve, *θ* is a degree of curvature, and *ETR*_max_ is the maximum *ETR*. Because the first derivative of Equation 18 is always greater than zero, we cannot use Equation 18 to estimate *I*_sat_.

Similarly, combing Equation 18 with *ETR* = *α*×*β*×Φ_PSII_×*I* (Krall and Edwards, 1992), we get the following expression for Φ_PSII_:


(19) $${\text{Φ}}_{\text{PSII}}=\frac{\alpha^{\prime }\times I+ETR_{\text{max}}-\sqrt{{\left(\alpha^{\prime }\times I+ETR_{\text{max}}\right)}^{2}-4\theta \times \alpha^{\prime }\times I\times ETR_{\text{max}}}}{2\theta \times \alpha \times \beta \times I}$$

Equations 18 and 19 represent how the NRH model (Model 4) describes *ETR*–*I* and Φ_PSII_–*I* curves. However, it should be noted that Φ_PSIImax_ and its corresponding *I*_sat_ cannot be estimated by Equation 19. In addition, there are two values of *ETR*_max_ to be returned when both *ETR*–*I* and Φ_PSII_–*I* curves are simulated by Equations 18 and 19, respectively.

### Chlorophyll a fluorescence measurement

Measurements were performed on plant leaves using a LI-6800 portable photosynthesis system (LI-COR Inc., USA) equipped with a LI-6800-01A leaf chamber fluorometer (LI-COR Inc., USA). A fully unfolded, dark green, and healthy leaf was used for each measurement. The initial fluorescence (*F*_0_) was recorded after 25 min of dark adaptation in the cuvette. The maximal fluorescence level of the dark-adapted leaves (*F*_m_) and light-adapted leaves (*F*_m_′) were determined by applying saturating flashes (15,000 μmol photons m^−2^ s^−1^) lasting 1 s, to promote the closure of the PSII reaction centers (Maxwell and Johnson, 2000). *F*_v_/*F*_m_ and *NPQ* were calculated as (*F*_m_−*F*_0_)/*F*_m_ and (*F*_m_−*F*_m_′)/*F*_m_′, respectively (van Kooten and Snel, 1990).

Light response measurements were conducted on sunny days from 8:30–11:30 a.m. to 2:00–5:00 p.m. using the automatic measurement program of the LI-6800 system. Leaves were flatly clamped into the leaf chamber and gradually exposed to light intensities of 0 μmol photons m^−2^ s^−1^, 25 μmol photons m^−2^ s^−1^, 50 μmol photons m^−2^ s^−1^, 100 μmol photons m^−2^ s^−1^, 200 μmol photons m^−2^ s^−1^, 300 μmol photons m^−2^ s^−1^, 400 μmol photons m^−2^ s^−1^, 600 μmol photons m^−2^ s^−1^, 800 μmol photons m^−2^ s^−1^, 1,000 μmol photons m^−2^ s^−1^, 1,200 μmol photons m^−2^ s^−1^, 1,400 μmol photons m^−2^ s^−1^, 1,600 μmol photons m^−2^ s^−1^, 1,800 μmol photons m^−2^ s^−1^, 1,900 μmol photons m^−2^ s^−1^, to 2,000 μmol photons m^−2^ s^−1^. For each light intensity, a minimum waiting time of 2 min and a maximum waiting time of 3 min were set before recording data. The instrument automatically matched the reference and sample chambers before data recording to ensure accuracy. The ambient CO_2_ concentration in the leaf chamber was maintained at 410 μmol mol^−1^, supplied via an external CO_2_ gas cylinder connected to the instrument’s CO_2_ injection system, with a flowrate of 500 μmol s^−1^. Air temperature in the leaf chamber was set at 30°C. Before measurements, leaves were exposed to sunlight or a light intensity of 1,800 μmol photons m^−2^ s^−1^ for 40 min to ensure activation. *ETR* was calculated as *ETR* = *α*×*β*×Φ_PSII_×*I*, where *α* is the distribution coefficient of absorption light energy by PSII and PSI, assumed to be 0.5 (Krall and Edwards, 1992; Maxwell and Johnson, 2000; Evans, 2009), and *β* is leaf absorptance, assumed to be 0.84 (Ehleringer, 1981). In this study, the values of Φ_PSII_ at *I* = 0 μmol photons m^−2^ s^−1^ were taken as the maximum Φ_PSII_ (Φ_PSIImax_). Key parameters (e.g., Φ_PSIImax_, *ETR*_max_, *F*_v_/*F*_m_, and *I*_sat_) from *ETR*–*I* curves and Φ_PSII_–*I* curves were fitted using Model 1–4, respectively, with SPSS 24.0 statistical software (SPSS, Chicago, IL).

### Chlorophyll content

Leaf disks were removed from the labeled leaves, followed by rapidly clipping of leaf area of 1 cm diameter for each leaf, to be cut into fine shreds and placed into glass test tube containing 5 mL of 80% (v/v) acetone. The airtight tubes were placed in the dark overnight or until the leaf was blanched at 25°C. All treatments were performed in triplicate. The extracts were centrifuged at 4,000 rpm for 10 min. Absorbances at 663 nm and 645 nm were measured using a spectrophotometer (UVICON-930, Kontron Instruments, Zürich, Switzerland) to determine the contents of chlorophyll (Chl) *a* and Chl *b* according to previous reported method by Wellburn (1994) (Wellburn, 1994). In addition, we may use the measured chlorophyll content to estimate *N*_0_ and then use Equation 1 to simulate the *ETR*–*I* curves of leaves to obtain *α*_e_, *β*_e_, and *γ*_e_, respectively. The values of *σ*_ik_ can be estimated by $\alpha_{\text{e}}=\frac{\alpha \beta N_{0}\sigma_{\text{ik}}\varphi }{S}$ (in this study, *α* is 0.5, *β* is 0.84, *φ* is 0.95, and *S* is 6×10^−4^ m^2^), the values of *σ*′_ik_ can be estimated by Equation 7 when the values of *σ*_ik_, *β*_e_, and *γ*_e_ were determined. *ETR*–*I* curves were fitted with the Photosynthesis Model Simulation Software (PMSS) at http://photosynthetic.sinaapp.com/index.html, in both Chinese and English, using Simulated Annealing and the Metropolis Algorithm to extract key parameters (e.g., *α*_e_, *β*_e_, *γ*_e_, *σ*_ik_, *σ′*_ik_, *ETR*_max_, and *I*_sat_).

### Statistical analyses

All variables are expressed as mean values ± *SE* from five samples for each species. Data were analyzed with one-way analysis of variance (ANOVA), and then, the values of *ETR*_max_ and *I*_sat_ estimated by four models were compared using a paired-sample *t*-test at *p* < 0.05 (*p*-significance level) using the SPSS 24.0 statistical software (SPSS, Chicago, IL). In addition, to compare the advantages and disadvantages of the study models, we took the Akaike’s information criterion (*AIC*), mean absolute error (MAE), and determination coefficient (*R* ^2^) as indicators to assess the fitting results of the three models. *AIC* was calculated by reference to Akaike’s method (Akaike, 1974), which equals 2*k*+*n*×ln(*SSR*/*n*) (here, *k* is the number of parameters, *n* is the sample size, and *SSR* is the sum square of residuals) and *R* ^2^ was given directly by SPSS 24.0 after fitting the data.

## Results

### Light response curves of *ETR*

**Figure 1:**
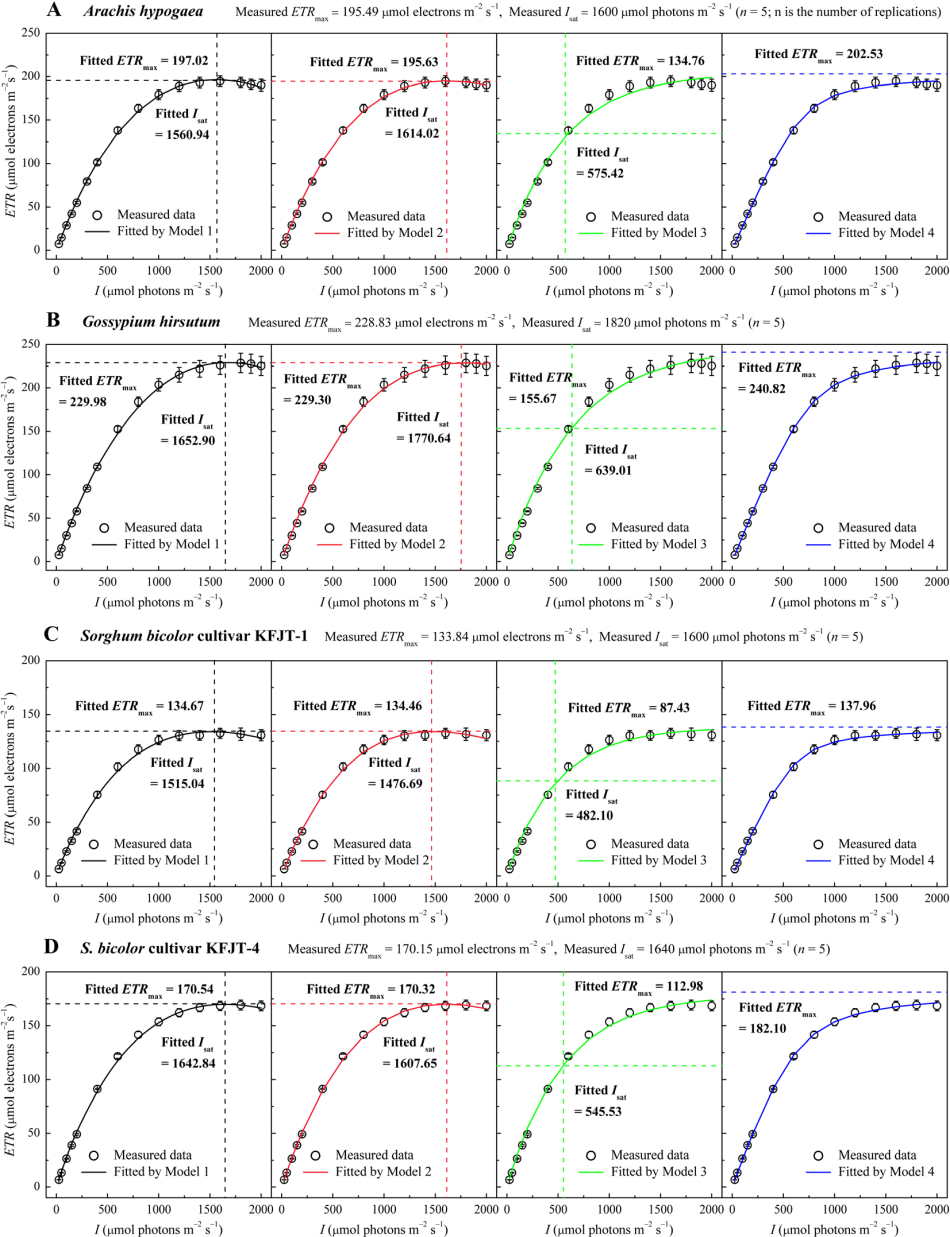
Light response curves of the electron transport rate (*ETR*–*I*) for various crops—*Arachis hypogaea* **(A)**, *Gossypium hirsutum* **(B)**, *Sorghum bicolor* cultivar KFJT-1 **(C)**, and *S. bicolor* cultivar KFJT-4 **(D)**. The curves were simulated by Models 1–4, respectively. Values were presented as means ± *SE* (*n* = 5). A horizontal dashed line represents the the fitted value of *ETR*_max_ from the model (μmol electrons m^−2^ s^−1^), while a vertical dashed line represents the fitted value of *I*_sat_ from the model (μmol photons m^−2^ s^−1^).

All plant species exhibited a characteristic rapid initial increase in *ETR* with rising *I*, followed by a saturation phase (**Figure 1**). Both C_3_ crops (*A. hypogaea* and *G. hirsutum*) displayed a slight decline in *ETR* beyond the saturation *I*, indicating a dynamic downregulation of PSII or photoinhibition (**Figures 1A, B**). The observed values of *I*_sat_ were approximately 1,600 μmol photons m^−2^ s^−1^and 1,820 μmol photons m^−2^ s^−1^ for *A. hypogaea* and *G. hirsutum*, respectively, with the corresponding *ETR*_max_ values as approximately 195.49 µmol electrons m^–2^ s^–1^ and 228.83 µmol electrons m^–2^ s^–1^ (**Figures 1A, B**; **Table 2**). In contrast, the two cultivars of the C_4_ crop *S. bicolor* showed less notable reduction in *ETR* after *I* surpassed *I*_sat_ (**Figures 1C, D**). The observed values of *ETR*_max_ for KFJT-1 and KFJT-4 were approximately 133.84 µmol electrons m^–2^ s^–1^ and 170.15 µmol electrons m^–2^ s^–1^, respectively, with the corresponding *I*_sat_ values approximately 1,600 μmol photons m^−2^ s^−1^ (**Figures 1C, D**; **Table 3**).

**Table 2:** Fitted (Equations 2, 13, 15, 19) and measured (Obs.) values of parameters defining *ETR–I* curves of *A. hypogaea* and *G. hirsutum*.

Parameters defining ETR–I curves	*A. hypogaea*					*G. hirsutum*				
	Model 1 (Equation 2)	Model 2 (Equation 13)	Model 3 (Equation 15)	Model 4 (Equation 19)	Obs.	Model 1 (Equation 2)	Model 2 (Equation 13)	Model 3 (Equation 15)	Model 4 (Equation 19)	Obs.
*I*_sat_	1,560.94 ± 23.41^a^	1,614.02 ± 46.45^a^	575.42 ± 23.93^b^	―	1,600.00 ± 63.24^a^	1,652.90 ± 21.40^b^	1,770.64 ± 62.61^a^	639.01 ± 33.61^c^	―	1,820.00 ± 20.00^a^
*ETR*_max_	197.02 ± 6.41^a^	195.63 ± 6.11^a^	134.76 ± 5.28^b^	202.53 ± 6.21^a^	195.49 ± 5.81^a^	229.98 ± 10.76^a^	229.30 ± 11.16^a^	155.67 ± 8.99^b^	240.82 ± 12.32^a^	228.83 ± 11.00^a^
*Φ*_PSIImax_	―	0.656 ± 0.005	―	―	―	―	0.750 ± 0.069	―	―	―
*F*_v_ */F*_m_	―	―	0.709 ± 0.026^a^	―	0.726 ± 0.003^a^	―	―	0.729 ± 0.033^a^	―	0.722 ± 0.006^a^
*R* ^2^	0.9991 ± 0.0002	0.9988 ± 0.0003	0.9929 ± 0.0008	0.9987 ± 0.0003	―	0.9989 ± 0.0001	0.9986 ± 0.0002	0.9945 ± 0.0001	0.9997 ± 0.0001	―
MAE	1.76 ± 0.30	2.00 ± 0.34	7.84 ± 2.59	2.09 ± 0.19	―	2.37 ± 0.10	2.67 ± 0.28	5.58 ± 0.24	1.12 ± 0.05	―
Number of parameters	3	4	4	3	―	3	4	4	3	―
*AIC*	19.08	23.05	32.28	21.19	―	21.82	25.52	32.20	13.30	―

The parameters are the saturation *I* (*I*_sat_, μmol photons m^−2^ s^−1^) and the maximum electron transport rate (*ETR*_max_, μmol electrons m^−2^ s^−1^). All values are the means ± *SE* (*n* = 5). Different letters denote statistically significant differences (*p*<0.05) between each row of fitted (Equations 2, 13, 15, 19) and measured (Obs.) values for each species. See **Table 1** for definitions of abbreviations.

**Table 3:** Fitted (Equations 2, 13, 15, 19) and measured (Obs.) values of parameters defining *ETR–I* curves of two *S. bicolor* cultivars.

Parameters defining *ETR–I* curves	*S. bicolor* KFJT-1					*S. bicolor* KFJT-4				
	Model 1 (Equation 2)	Model 2 (Equation 13)	Model 3 (Equation 15)	Model 4 (Equation 19)	Obs.	Model 1 (Equation 2)	Model 2 (Equation 13)	Model 3 (Equation 15)	Model 4 (Equation 19)	Obs.
*I*_sat_	1,515.04 ± 33.30^a^	1,476.69 ± 33.56^a^	482.10 ± 18.91^b^	―	1,600.00 ± 63.24^a^	1,642.84 ± 29.62^a^	1607.65 ± 37.93^a^	545.53 ± 20.00^b^	―	1,640.00 ± 74.83^a^
*ETR*_max_	134.67 ± 5.05^a^	134.46 ± 4.96^a^	87.43 ± 3.45^b^	137.96 ± 5.46^a^	133.84 ± 5.52^a^	170.54 ± 4.40^b^	170.32 ± 4.33^b^	112.98 ± 3.44^c^	182.10 ± 4.97^a^	170.15 ± 4.45^b^
*Φ*_PSIImax_	―	0.672 ± 0.062	―	―	―	―	0.674 ± 0.028	―	―	―
*F*_v_ */F*_m_	―	―	0.675 ± 0.032^a^	―	0.688 ± 0.004^a^	―	―	0.672 ± 0.038^a^	―	0.714 ± 0.001^a^
*R* ^2^	0.9979 ± 0.0005	0.9976 ± 0.0008	0.9935 ± 0.0009	0.9985 ± 0.0003	―	0.9991 ± 0.0001	0.9991 ± 0.0002	0.9968 ± 0.0005	0.9994 ± 0.0002	―
MAE	1.90 ± 0.26	1.81 ± 0.23	3.47 ± 0.30	1.65 ± 0.19	―	1.61 ± 0.07	1.51 ± 0.09	3.20 ± 0.28	1.24 ± 0.17	―
Number of parameters	3	4	4	3	―	3	4	4	3	―
*AIC*	19.51	22.27	27.05	14.50	―	17.51	19.44	25.90	13.28	―

The parameters are the saturation *I* (*I*_sat_, μmol photons m^−2^ s^−1^) and the maximum electron transport rate (*ETR*_max_, μmol electrons m^−2^ s^−1^). All values are the means ± *SE* (*n* = 5). Different letters denote statistically significant differences (*p*<0.05) between each row of fitted (Equations 2, 13, 15, 19) and measured (Obs.) values for each species. See **Table 1** for definitions of abbreviations.

Compared to Models 1, 2, and 4, Model 3 largely failed to represent the observed *ETR–I* curves (**Figure 1**). Models 1, 2, and 4 demonstrated high goodness-of-fit, based on the coefficient of determination (*R* ^2^) and mean absolute error (MAE) (**Tables 2**, **3**). Although Model 4 showed higher *R* ^2^ values than Models 1 and 2 (**Tables 2**, **3**), it overestimated *ETR*_max_ and cannot return *I*_sat_. Models 1 and 2 provided *ETR*_max_ and *I*_sat_ values closely aligned with observed data across all species, but Model 1 demonstrated the lowest *AIC*, indicating an optimal balance between predictive accuracy and model parsimony. Model 3 underestimated both *ETR*_max_ and *I*_sat_, with significant differences between fitted and measured data across all crops (*p*<0.05) (**Figure 1C**; **Tables 2**, **3**). Additionally, despite Model 3 being able to produce *F*_v_/*F*_m_ values by fitting *ETR–I* curves, these values significantly deviated from the observed *F*_v_/*F*_m_ across all crops.

### Light response curves of Φ_PSII_

**Figure 2:**
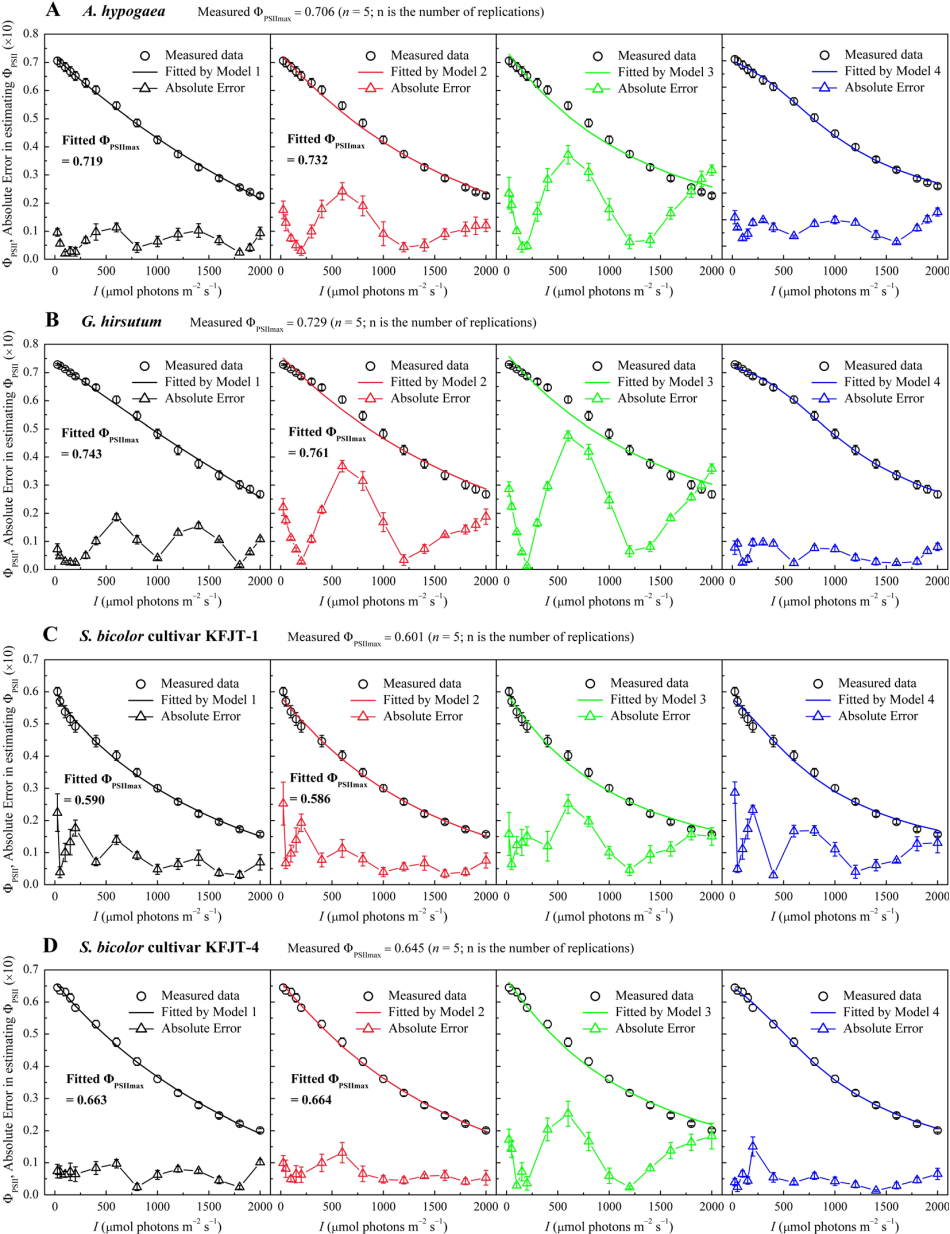
Light response curves of effective quantum efficiency (Φ_PSII_–*I*) for various crops—*A. hypogaea* **(A)**, *G. hirsutum* **(B)**, *S. bicolor* cultivar KFJT-1 **(C)**, and *S. bicolor* cultivar KFJT-4 **(D)**. The curves were simulated by Model 1–4, respectively, and the fitted absolute error is shown. Values were presented as means ± *SE* (*n* = 5).

The four models varied significantly in characterizing Φ_PSII_ *–I* curves (**Figure 2**; **Tables 4**, **5**). The Φ_PSII_ *–I* curves, fitted using Models 1 (Equation 6), 2 (Equation 14), 3 (Equation 16), and 4 (Equation 18), exhibited a characteristic decrease in Φ_PSII_ with the increasing *I* for all crops (**Figure 2**). Among the models, Model 1 most accurately simulated the nonlinear relationship between Φ_PSII_ and *I*, obtaining the highest *R* ^2^ and the lowest MAE compared to Models 2 and 3, which exhibited notable deviation from observations, particularly for *A. hypogaea* and *G. hirsutum* (**Figures 2A, B**; **Tables 4**, **5**). Despite being able to estimate *F*_v_ */F*_m_, Model 3 produced *F*_v_/*F*_m_ values that were significantly different from the measured values. Additionally, Model 3 generated an *I*_sat_ value with an unknown or unclear meaning (**Tables 4**, **5**). While Model 4 exhibited the highest fitting degree for *G. hirsutum* and *S. bicolor* KFJT-4, the fitted curves fluctuated in the low light intensity range (below 600 μmol photons m^−2^ s^−1^) (**Figure 2**). Furthermore, Model 4 generated a significantly higher *ETR*_max_ than the measured value. Another key difference among the models is their ability to return the Φ_PSIImax_. Models 1 and 2 can return Φ_PSIImax_, while Models 3 and 4 cannot. Compared to Model 2, Model 1 returned Φ_PSIImax_ values closer to the observed values.

**Table 4:** Fitted (Equations 6, 14, 16, 18) and measured (Obs.) values of parameters defining Φ_PSII_–*I* curves of *A. hypogaea* and *G. hirsutum.*.

Parameters defining Φ_PSII_–*I* curves	*A. hypogaea*					*G. hirsutum*				
	Model 1 (Equation 6)	Model 2 (Equation 14)	Model 3 (Equation 16)	Model 4 (Equation 18)	Obs.	Model 1 (Equation 6)	Model 2 (Equation 14)	Model 3 (Equation 16)	Model 4 (Equation 18)	Obs.
*Φ*_PSIImax_	0.719 ± 0.016^a^	0.732 ± 0.017^a^	―	―	0.706 ± 0.012^a^	0.743 ± 0.004^b^	0.761 ± 0.006^a^	―	―	0.729 ± 0.003^b^
*I*_sat_	―	―	747.37 ± 35.72	―	―	―	―	883.75 ± 55.85	―	―
*F*_v_ */F*_m_	―	―	0.742 ± 0.018^a^	―	0.726 ± 0.003^a^	―	―	0.769 ± 0.005^a^	―	0.722 ± 0.006^b^
*ETR*_max_	―	―	―	212.73 ± 10.49	―	―	―	―	235.55 ± 6.96	―
*R* ^2^	0.9981 ± 0.0003	0.9939 ± 0.0017	0.9836 ± 0.0026	0.9913 ± 0.0060	―	0.9969 ± 0.0003	0.9877 ± 0.0026	0.9763 ± 0.0028	0.9985 ± 0.0002	―
MAE	0.0064 ± 0.0008	0.0112 ± 0.0015	0.0192 ± 0.0016	0.0074 ± 0.0007	―	0.0078 ± 0.0005	0.0156 ± 0.0012	0.0223 ± 0.0008	0.0060 ± 0.0005	―
Number of parameters	3	2	2	5	―	3	2	2	5	―
*AIC*	−36.60	−31.07	−26.06	−25.67	―	−34.79	−28.10	−24.72	−38.52	―

The parameters are the maximum effective quantum efficiency of PSII (Φ_PSIImax_), the saturation I (I_sat_, μmol photons m^−2^ s^−1^), the maximum quantum efficiency of PSII (*F*_v_/*F*_m_), and the maximum electron transport rate (*ETR*_max_, μmol electrons m^−2^ s^−1^). All values are the means ± *SE* (*n* = 5). Different letters denote statistically significant differences (*p*< 0.05) between each row of fitted (Equations 6, 14, 16, 18) and measured (Obs.) values for each light environment. See **Table 1** for definitions of abbreviations.

**Table 5:** Fitted (Equations 6, 14, 16, 18) and measured (Obs.) values of parameters defining Φ_PSII_–*I* curves of two *S. bicolor* cultivars.

Parameters defining Φ_PSII_–*I* curves	*S. bicolor* KFJT-1					*S. bicolor* KFJT-4				
	Model 1 (Equation 6)	Model 2 (Equation 14)	Model 3 (Equation 16)	Model 4 (Equation 18)	Obs.	Model 1 (Equation 6)	Model 2 (Equation 14)	Model 3 (Equation 16)	Model 4 (Equation 18)	Obs.
*Φ*_PSIImax_	0.590 ± 0.018^a^	0.586 ± 0.019^a^	―	―	0.601 ± 0.013^a^	0.663 ± 0.011^a^	0.664 ± 0.013^a^	―	―	0.645 ± 0.010^b^
*I*_sat_	―	―	597.87 ± 24.17	―	―	―	―	687.56 ± 23.40	―	―
*F*_v_ */F*_m_	―	―	0.598 ± 0.019^b^	―	0.688 ± 0.004^a^	―	―	0.675 ± 0.013^b^	―	0.714 ± 0.001^a^
*ETR*_max_	―	―	―	144.55 ± 7.06	―	―	―	―	177.29 ± 7.90	―
*R* ^2^	0.9932 ± 0.0023	0.9926 ± 0.0025	0.9885 ± 0.0023	0.9894 ± 0.0031	―	0.9978 ± 0.0004	0.9973 ± 0.0004	0.9915 ± 0.0019	0.9983 ± 0.0005	―
MAE	0.0093 ± 0.0009	0.0095 ± 0.0009	0.0133 ± 0.0009	0.0126 ± 0.0014	―	0.0067 ± 0.0009	0.0069 ± 0.0008	0.0123 ± 0.0020	0.0050 ± 0.0009	―
Number of parameters	3	2	2	5	―	3	2	2	5	―
*AIC*	−33.12	−34.68	−32.26	−26.86	―	−36.22	−38.53	−32.73	−35.12	―

The parameters are the maximum effective quantum efficiency of PSII (Φ_PSIImax_), the saturation *I* (*I*_sat_, μmol photons m^−2^ s^−1^), the maximum quantum efficiency of PSII (*F*_v_/*F*_m_), and the maximum electron transport rate (*ETR*_max_, μmol electrons m^−2^ s^−1^). All values are the means ± *SE* (*n* = 5). Different letters denote statistically significant differences (*p*< 0.05) between each row of fitted (Equations 6, 14, 16, 18) and measured (Obs.) values for each light environment. See **Table 1** for definitions of abbreviations.

### Light response curves of *NPQ*, *N*_k_ and *σ*′_ik_

**Figure 3:**
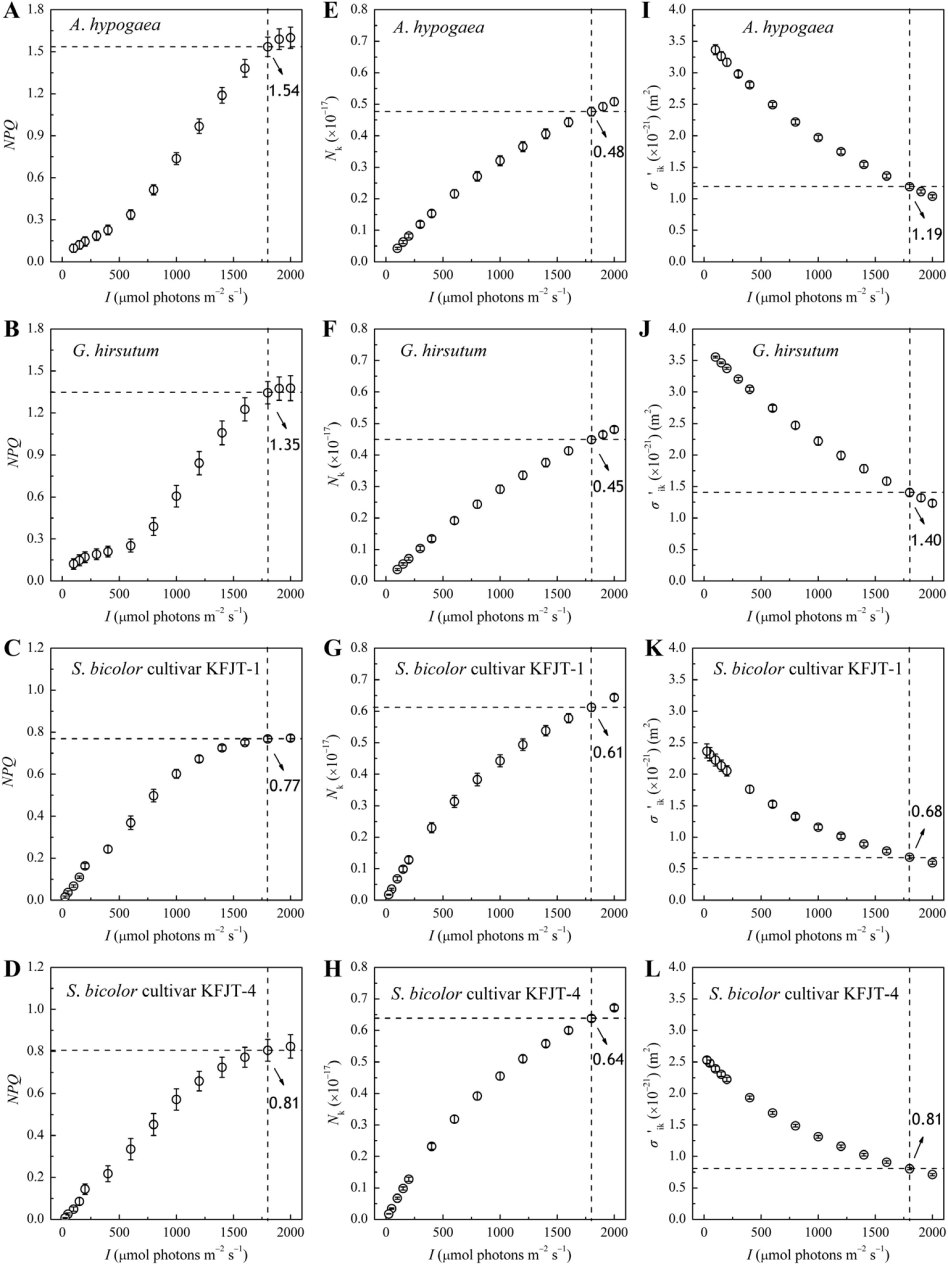
Non-photochemical quenching (*NPQ*), total light-harvesting pigment molecules in excited state (*N*_k_), and effective light energy absorption cross-section (*σ′*_ik_, m^2^) for *A. hypogaea* **(A, E, I)**, *G. hirsutum* **(B, F, J)**, *S. bicolor* cultivar KFJT-1 **(C, G, K)**, and *S. bicolor* cultivar KFJT-4 **(D, H, K)**. Values were presented as means ± *SE* (*n* = 5). The intersection of the black horizontal and vertical dashed lines in each graph represents their measured value at *I* = 1,800 μmol photons m^−2^ s^−1^.

*NPQ* increased nonlinearly with *I* across all plant species, with distinct patterns observed between C_3_ and C_4_ plants (**Figures 3A–D**). When *I* was below 600 μmol photons m^−2^ s^−1^, the *NPQ* of *A. hypogaea* and *G. hirsutum* increased very slowly with the increasing *I*. When *I* was more than 600 μmol photons m^−2^ s^−1^, *NPQ* increased rapidly (**Figures 3A–D**). In contrast, the *NPQ* of the two *S. bicolor* cultivars showed nearly linear increase when *I* was below 1,800 μmol photons m^−2^ s^−1^. Under high *I* condition, the *NPQ* of *A. hypogaea* and *G. hirsutum* was significantly higher than that of the two *S. bicolor* cultivars. For example, at 1,800 μmol photons m^−2^ s^−1^, the *NPQ* values of *A. hypogaea* and *G. hirsutum* were 1.54 and 1.35, respectively, whereas the *NPQ* values of the two *S. bicolor* cultivars were only 0.77 and 0.81 (**Figures 3A–D**). *N*_k_ increased nonlinearly with the increasing *I* for all crops (**Figure 3E–H**), more rapidly in *S. bicolor* compared to that in *A. hypogaea* and *G. hirsutum*. At *I* = 1,800 μmol photons m^−2^ s^−1^, the *N*_k_ values for *S. bicolor* cultivars KFJT-1 and KFJT-4 were 0.61 and 0.64, respectively, and *A. hypogaea* and *G. hirsutum* demonstrated lower *N*_k_ values (0.48 and 0.45, respectively).

During photosynthesis, light-harvesting pigment molecules absorb light energy and transition to different excited states. The ability of these molecules to absorb light energy is represented by their effective absorption cross-section (*σ′*_ik_). As illustrated in **Figures 3I–L**, *σ′*_ik_ nonlinearly decreased with the increasing *I* for all crops, whose *σ′*_ik_ *–I* curves exhibited similar trends to their Φ_PSII_ *–I* curves (**Figure 2**), indicating a strong correlation between light absorption and photosynthetic efficiency. Within the tested range of *I*, *A. hypogaea* and *G. hirsutum* exhibited significantly higher *σ′*_ik_ values compared to the two cultivars of *S. bicolor*. When *I* was 1,800 μmol m^-2^ s^-1^, the *σ′*_ik_ of *A. hypogaea* and *G. hirsutum* were 1.19 × 10^-21^ m^2^ and 1.40 × 10^-21^ m^2^, respectively (**Figures 3I, J**), and the *σ′*_ik_ values of *S. bicolor* cultivars KFJT-1 and KFJT-4 were 0.68 × 10^-21^ m^2^ and 0.81 × 10^-21^ m^2^ (**Figures 3K, L**). *σ*_ik_ showed similar inter-specific difference as that of *σ′*_ik_. The *σ*_ik_ values estimated by Model 1 (Equation 1) for *A. hypogaea* and *G. hirsutum* were (3.60 ± 0.10) × 10^−21^ m^2^ and (3.74 ± 0.23) × 10^−21^ m^2^, respectively. The *σ*_ik_ values of *S. bicolor* cultivars KFJT-1 and KFJT-4 were (2.42 ± 0.12) × 10^−21^ m^2^ and (2.58 ± 0.54) × 10^−21^ m^2^, respectively.

## Discussion

### Empirical models and biological integration

Classic empirical models typically rely on mathematical analyses of measured data to establish quantitative functions, often lacking explicit incorporation of biological processes. Through the intercomparison among the four models and observations, this study demonstrates that Model 1 (Ye model) can accurately and simultaneously simulate *ETR*–*I* and Φ_PSII_–*I* curves. Model 1 demonstrated its consistent robustness and accuracy for the studied crops in (1) reproducing the *ETR*–*I* and Φ_PSII_–*I* curves and (2) returning key quantitative traits defining the light response functions.

By employing an explicit and transparent analytical framework with consistent definitions, Ye model incorporates the fundamental processes of light energy absorption, conversion, and transfer to the reaction centers of PSII via photosynthetic pigments. These processes include light harvesting, exciton resonance transfer, quantum level transitions, and de-excitation (Ye et al., 2013a, 2013b; Shevela et al., 2023). Equation 5 incorporated the quantitative relationship between Φ_PSII_ and the intrinsic characteristics of light-harvesting pigment molecules (i.e., *N*_0_, *σ*_ik_, *τ*, *φ*, *k*_P_, *k*_D_, *g*_i_, *g*_k_, *ξ*_1_, *ξ*_2_, and *ξ*_3_. Our results highlight that the consistent decrease in Φ_PSII_ and *σ′*_ik_ with the increasing *I* (**Figures 2**, **3I–L**), a finding consistent with previous studies (Suggett et al., 2004, 2007; Ye et al., 2013a).

### Validation of model predictions

The observed decrease in *σ′*_ik_ with increasing *I* (**Figures 3I–L**) support previous studies (Suggett et al., 2004, 2007; Ye et al., 2013a). For instance, Suggett et al. (2004) reported the increase in effective absorption cross-sections for PSII of *Emiliania huxleyi* with the decrease in *I* in the plant growth environment. These results demonstrated that plants could adjust their light absorption properties to optimize photosynthetic efficiency and minimize photodamage under the changing light environment.

Moreover, the values of *ETR*_max_ and *I*_sat_ fitted by Ye model (Equation 2) were in close agreement with the observed data (**Figure 1**; **Tables 2**, **3**), supporting previous reports (Ye et al., 2013a, 2013b; Robakowski et al., 2018). In contrast, Model 3 underestimated *ETR*_max_ and the corresponding *I*_sat_ (**Figure 1**; **Tables 2**, **3**). While Model 4 has been widely used to estimate *ETR*_max_ and is a sub-model of FvCB model (Farquhar et al., 1980; von Caemmerer, 2000; Long and Bernacchi, 2003; Sharkey et al., 2007; Miao et al., 2009; Yin et al., 2009; Gu et al., 2010; Bernacchi et al., 2013; von Caemmerer, 2013; Park et al., 2016; Cai et al., 2018; Yin et al., 2021), it overestimated *ETR*_max_ and cannot return the corresponding *I*_sat_ (**Figure 1**; **Tables 2**, **3**). These results support the previous studies reporting the limitations of these empirical models in accurately characterizing *ETR*–*I* curves (Smyth et al., 2004; Silsbe and Kromkamp, 2012; Buckley and Diaz‐Espejo, 2015; Yang et al., 2024). Meanwhile, Ye model (Equation 6) can also accurately characterize the Φ_PSII_ *–I* curves (**Figure 2**; **Tables 4**, **5**). The negative exponential function (Model 2) overestimated Φ_PSIImax_ (**Figure 2**; **Tables 4**). The exponential function (Model 3) and the NRH model (Model 4) cannot return Φ_PSIImax_ (**Tables 4**, **5**).

### Photosynthetic differences between C_3_ and C_4_ plants

Our study also highlights distinct photosynthetic responses between C_3_ and C_4_ plants. For instance, the Φ_PSII_ in *A. hypogaea* and *G. hirsutum* was significantly higher than that in two cultivars of *S. bicolor*, suggesting that C_3_ plants may possess a greater capacity for light energy utilization compared to C_4_ plants. The results demonstrate that Ye model (Model 1) can be used to estimate the absorption cross-section of pigment molecules for both C_3_ and C_4_ plants, supporting previous studies on other photosynthetic organisms (e.g., algae and cyanobacteria) (Yang et al., 2023; Ye et al., 2024). Ley and Mauzerall (1982) (Ley and Mauzerall, 1982) reported that the absolute absorption cross-section for oxygen production for chlorophyll in *Chlorella vulgaris* at 596 nm *in vivo* was 2.9×10^–21^ m^2^, which was independent of total cell chlorophyll content. In this study, *σ*_ik_ estimated by Ye model (Equation 1) for *A. hypogaea*, *G. hirsutum*, *S. bicolor* KFJT-1, and *S. bicolor* KFJT-4 were 3.60 × 10^−21^ m^2^, 3.74 × 10^−21^ m^2^, 2.42 × 10^−21^ m^2^ and 2.58 × 10^−21^ m^2^, respectively.

C_3_ and C_4_ plants represent distinct evolutionary adaptations to different environmental conditions. The differences in their photosynthetic machinery and carbon fixation mechanisms lead to distinct responses in parameters such as Φ_PSII_, *ETR*, and *NPQ* across light intensities (Stefanov et al., 2022). The different *σ*_ik_ values between C_3_ and C_4_ plants observed in this study reflect their differential adaptations to light environments. C_3_ plants, which evolved in more moderate light environments, typically have a higher light absorption capacity (i.e., higher *σ*_ik_). It allows them to efficiently capture light in potentially light-limited conditions. However, this higher absorption capacity also makes them more susceptible to photoinhibition at high light intensities, as observed in the *ETR–I* curves where *A. hypogaea* and *G. hirsutum* showed more notable decline in *ETR* beyond *I*_sat_ (**Figure 1**). In contrast, the C_4_ plants (*S. bicolor*) in this study showed lower *σ*_ik_ values, contributing to their ability to maintain relatively constant *ETR* even when *I* surpasses *I*_sat_ (**Figure 1**). The carbon-concentrating mechanism allows C_4_ plants to maintain high photosynthetic rates under high light conditions without the need for excessive light absorption, thereby reducing the risk of photodamage (Yang et al., 2024).

### Model limitations and implications

While the Ye model provides a robust representation of light response mechanisms in PSII, it has limitations that warrant further research. The model assumes steady-state photosynthetic conditions, which may not fully capture dynamic processes such as stomatal closure, changes in chloroplast morphology, or fluctuating light intensities. Additionally, factors such as nutrient availability, water stress, or temperature effects are not explicitly included in the current framework. These variables can significantly impact photosynthetic efficiency and could be integrated into future iterations of the model. This study validated the model on a limited set of crops (peanut, cotton, and sweet sorghum) under controlled conditions. Therefore, future studies should expand validation to a broader range of species (e.g., maize, rice, and wheat), and environmental contexts (e.g., fluctuating light, drought, and extreme temperatures) would strengthen the generalizability of model. Incorporating dynamic elements into the model to simulate transient responses to light fluctuations would be valuable. Furthermore, investigating the integration of abiotic stress factors into the model framework could improve predictions of photosynthetic efficiency under stress conditions such as drought or extreme heat.

The accurate assessment of key photosynthetic parameters, such as *ETR*_max_, Φ_PSIImax_, *I*_sat_, *σ*_ik_, *σ*′_ik_, *N*_k_, and *NPQ*, positions the Ye model as a transformative tool for advancing photosynthetic research. These parameters are critical in understanding how plants respond to fluctuating light environments, optimize photochemical efficiency, and manage photoprotection. For instance, the ability of Ye model to quantify *N*_k_ and *σ*′_ik_ enables detailed exploration of how light-harvesting complexes dynamically adjust to varying light intensities. Similarly, the coupling of Φ_PSII_ and *NPQ* predictions provides insight into the balance between photochemical utilization and dissipation of excess light energy, a critical factor under high-stress conditions such as drought or extreme light fluctuations.

This capability has direct implications for plant breeding and crop management in the context of climate change. By leveraging the outputs of the model, researchers can identify genotypes with optimized light absorption and photoprotective traits for high light variability scenarios, such as those experienced in marginal or degraded agricultural lands. Moreover, the model can support breeding programs aimed at developing cultivars with enhanced yield stability by selecting for traits that mitigate photoinhibition or excessive *NPQ* under fluctuating light. Additionally, incorporating these parameter assessments into ecosystem and agricultural productivity models can improve predictions of carbon assimilation and crop yield under diverse environmental conditions.

To the best of our knowledge, this is the first study reporting a robust model (Equations 2, 6) that can simultaneously and accurately simulate *ETR–I* and Φ_PSII_ *–I* curves and returning values of key physical and biochemical parameters of photosynthetic pigments (i.e., intrinsic absorption cross-section and the effective absorption cross-section of light-harvesting pigment molecules). The findings could also help quantify key light-harvesting properties associated with photoacclimation (Fiebig et al., 2023), photoprotection (Niyogi and Truong, 2013), dynamic downregulation of PSII (Ralph and Gademann, 2005), and/or photoinhibition (Govindjee, 2002) in response to environmental change. This study is useful for (1) plant experimentalists quantifying intra- and/or inter-specific variation in Φ_PSII_–*I* responses and (2) modelers working on better model representation of photosynthetic processes under dynamic light environment.

## Data Availability

The original contributions presented in the study are included in the article/supplementary material. Further inquiries can be directed to the corresponding authors.

## References

[B1] Akaike H. (1974). A new look at the statistical model identification. IEEE T. Automat. Contr. 19, 716–723. doi: 10.1109/TAC.1974.1100705

[B2] Baker N. R. (2008). Chlorophyll fluorescence: a probe of photosynthesis *in vivo* . Annu. Rev. Plant Biol. 59, 89–113. doi: 10.1146/annurev.arplant.59.032607.092759 18444897

[B3] Bernacchi C. J. Bagley J. E. Serbin S. P. Ruiz-Vera U. M. Rosenthal D. M. Vanloocke A. (2013). Modelling C_3_ photosynthesis from the chloroplast to the ecosystem. Plant Cell Environ. 36, 1641–1657. doi: 10.1111/pce.2013.36.issue-9 23590343

[B4] Buckley T. N. Diaz-Espejo A. (2015). Reporting estimates of maximum potential electron transport rate. New Phytol. 205, 14–17. doi: 10.1111/nph.13018 25196056

[B5] Buckley T. N. Farquhar G. D. (2004). A new analytical model for whole-leaf potential electron transport rate. Plant Cell Environ. 27, 1487–1502. doi: 10.1111/j.1365-3040.2004.01232.x

[B6] Cai C. Li G. Yang H. Yang J. Liu H. Struik P. C.. (2018). Do all leaf photosynthesis parameters of rice acclimate to elevated CO_2_, elevated temperature, and their combination, in FACE environments? Global Change Biol. 24, 1685–1707. doi: 10.1111/gcb.13961 29076597

[B7] Córdoba J. Molina-Cano J. L. Martínez-Carrasco R. Morcuende R. Pérez P. (2016). Functional and transcriptional characterization of a barley mutant with impaired photosynthesis. Plant Sci. 244, 19–30. doi: 10.1016/j.plantsci.2015.12.006 26810450

[B8] Ehleringer J. J. O. (1981). Leaf absorptances of Mohave and Sonoran desert plants. Oecologia 49, 366–370. doi: 10.1007/BF00347600 28309998

[B9] Evans J. R. (2009). Potential errors in electron transport rates calculated from chlorophyll fluorescence as revealed by a multilayer leaf model. Plant Cell Physiol. 50, 698–706. doi: 10.1093/pcp/pcp041 19282373

[B10] Farquhar G. D. von Caemmerer S. Berry J. A. (1980). A biochemical model of photosynthetic CO_2_ assimilation in leaves of C_3_ species. Planta 149, 78–90. doi: 10.1007/BF00386231 24306196

[B11] Fiebig O. C. Harris D. Wang D. Hoffmann M. P. Schlau-Cohen G. S. (2023). Ultrafast dynamics of photosynthetic light harvesting: atrategies for acclimation across organisms. Ann. Rev. Phys. Chem. 74, 493–520. doi: 10.1146/annurev-physchem-083122-111318 36791782

[B12] Genty B. Briantais J.-M. Baker N. R. (1989). The relationship between the quantum yield of photosynthetic electron transport and quenching of chlorophyll fluorescence. BBA-Gen. Subj. 990, 87–92. doi: 10.1016/S0304-4165(89)80016-9

[B13] Govindjee (2002). A role for a light-harvesting antenna complex of photosystem II in photoprotection. Plant Cell 14, 1663–1668. doi: 10.1105/tpc.140810 12172013 PMC526029

[B14] Gu L. H. Pallardy S. G. Law B. E. Wullschleger S. D. (2010). Reliable estimation of biochemical parameters from C_3_ leaf photosynthesis-intercellular carbon dioxide response curves. Plant Cell Environ. 33, 1852–1874. doi: 10.1111/j.1365-3040.2010.02192.x 20561254

[B15] Krall J. P. Edwards G. E. (1992). Relationship between photosystem II activity and CO_2_ fixation in leaves. Physiol. Plantarum 86, 180–187. doi: 10.1111/j.1399-3054.1992.tb01328.x

[B16] Ley A. C. Mauzerall D. C. (1982). Absolute absorption cross-sections for photosystem II and the minimum quantum requirement for photosynthesis in *Chlorella vulgaris* . BBA-Bioenergetics 680, 95–106. doi: 10.1016/0005-2728(82)90320-6

[B17] Liu F. Song Q. Zhao J. Mao L. Bu H. Hu Y.. (2021). Canopy occupation volume as an indicator of canopy photosynthetic capacity. New Phytol. 232, 941–956. doi: 10.1111/nph.17611 34245568

[B18] Long S. P. Bernacchi C. J. (2003). Gas exchange measurements, what can they tell us about the underlying limitations to photosynthesis? Procedures and sources of error. J. Exp. Bot. 54, 2393–2401. doi: 10.1093/jxb/erg262 14512377

[B19] Maxwell K. Johnson G. N. (2000). Chlorophyll fluorescence—a practical guide. J. Exp. Bot. 51, 659–668. doi: 10.1093/jexbot/51.345.659 10938857

[B20] Miao Z. Xu M. Lathrop R. G. Jr. Wang Y. (2009). Comparison of the *A*–*C* _c_ curve fitting methods in determining maximum ribulose 1,5-bisphosphate carboxylase/oxygenase carboxylation rate, potential light saturated electron transport rate and leaf dark respiration. Plant Cell Environ. 32, 109–122. doi: 10.1111/j.1365-3040.2008.01900.x 19154228

[B21] Moin M. Bakshi A. Saha A. Udaya Kumar M. Reddy A. R. Rao K. V.. (2016). Activation tagging in indica rice identifies ribosomal proteins as potential targets for manipulation of water-use efficiency and abiotic stress tolerance in plants. Plant Cell Environ. 39, 2440–2459. doi: 10.1111/pce.12796 27411514

[B22] Niyogi K. K. Truong T. B. (2013). Evolution of flexible non-photochemical quenching mechanisms that regulate light harvesting in oxygenic photosynthesis. Curr. Opin. Plant Biol. 16, 307–314. doi: 10.1016/j.pbi.2013.03.011 23583332

[B23] Park K. S. Kim S. K. Cho Y.-Y. Cha M. K. Jung D. H. Son J. E. (2016). A coupled model of photosynthesis and stomatal conductance for the ice plant (*Mesembryanthemum crystallinum* L.), a facultative CAM plant. Hortic. Environ. Biote. 57, 259–265. doi: 10.1007/s13580-016-0027-7

[B24] Pavlovič A. Slováková L. U. Pandolfi C. Mancuso S. (2011). On the mechanism underlying photosynthetic limitation upon trigger hair irritation in the carnivorous plant Venus flytrap (*Dionaea muscipula* Ellis). J. Exp. Bot. 62, 1991–2000. doi: 10.1093/jxb/erq404 21289078 PMC3060689

[B25] Ralph P. J. Gademann R. (2005). Rapid light curves: a powerful tool assesses photosynthetic activity. Aquat. Bot. 82, 222–237. doi: 10.1016/j.aquabot.2005.02.006

[B26] Ritchie R. J. (2008). Fitting light saturation curves measured using modulated fluorometry. Photosynth. Res. 96, 201–215. doi: 10.1007/s11120-008-9300-7 18415696

[B27] Ritchie R. J. Bunthawin S. (2010). The use of pulse amplitude modulation (PAM) fluorometry to measure photosynthesis in a CAM orchid, *Dendrobium* spp. (D. cv. Viravuth Pink). Int. J. Plant Sci. 171, 575–585. doi: 10.1086/653131

[B28] Robakowski P. (2005). Susceptibility to low-temperature photoinhibition in three conifers differing in successional status. Tree Physiol. 25, 1151–1160. doi: 10.1093/treephys/25.9.1151 15996958

[B29] Robakowski P. Pers-Kamczyc E. Ratajczak E. Thomas P. A. Ye Z.-P. Rabska M.. (2018). Photochemistry and antioxidative capacity of female and male *Taxus baccata* L. acclimated to different nutritional environments. Front. Plant Sci. 9. doi: 10.3389/fpls.2018.00742 PMC5996056 29922316

[B30] Sharkey T. D. Bernacchi C. J. Fa Rquhar G. D. Singsaas E. L. (2007). Fitting photosynthetic carbon dioxide response curves for C_3_ leaves. Plant Cell Environ. 30, 1035–1040. doi: 10.1111/j.1365-3040.2007.01710.x 17661745

[B31] Shevela D. Kern J. F. Govindjee G. Messinger J. (2023). Solar energy conversion by photosystem II: principles and structures. Photosynth. Res. 156, 279–307. doi: 10.1007/s11120-022-00991-y 36826741 PMC10203033

[B32] Silsbe G. M. Kromkamp J. C. (2012). Modeling the irradiance dependency of the quantum efficiency of photosynthesis. Limnol. Oceanogr. - Meth. 10, 645–652. doi: 10.4319/lom.2012.10.645

[B33] Smyth T. J. Pemberton K. L. Aiken J. Geider R. J. (2004). A methodology to determine primary production and phytoplankton photosynthetic parameters from Fast Repetition Rate Fluorometry. J. Plankton Res. 26, 1337–1350. doi: 10.1093/plankt/fbh124

[B34] Song Q. Wang Y. Qu M. Ort D. R. Zhu X.-G. (2017). The impact of modifying photosystem antenna size on canopy photosynthetic efficiency—Development of a new canopy photosynthesis model scaling from metabolism to canopy level processes. Plant Cell Environ. 40, 2946–2957. doi: 10.1111/pce.13041 28755407 PMC5724688

[B35] Stefanov M. A. Rashkov G. D. Apostolova E. L. (2022). Assessment of the photosynthetic apparatus functions by chlorophyll fluorescence and P700 absorbance in C_3_ and C_4_ plants under physiological conditions and under salt stress. Int. J. Mol. Sci. 23, 3768. doi: 10.3390/ijms23073768 35409126 PMC8998893

[B36] Suggett D. J. Le Floc’H E. Harris G. N. Leonardos N. Geider R. J. (2007). Different strategies of photoacclimation by two strains of *Emiliania huxleyi* (Haptophyta). J. Phycol. 43, 1209–1222. doi: 10.1111/j.1529-8817.2007.00406.x

[B37] Suggett D. J. MacIntyre H. L. Geider R. J. (2004). Evaluation of biophysical and optical determinations of light absorption by photosystem II in phytoplankton. Limnol. Oceanogr. - Meth. 2, 316–332. doi: 10.4319/lom.2004.2.316

[B38] van der Tol C. Berry J. A. Campbell P. K. E. Rascher U. (2014). Models of fluorescence and photosynthesis for interpreting measurements of solar-induced chlorophyll fluorescence. J. Geophys. Res.-Biogeo. 119, 2312–2327. doi: 10.1002/2014JG002713 PMC4852699 27398266

[B39] van Kooten O. Snel J. F. H. (1990). The use of chlorophyll fluorescence nomenclature in plant stress physiology. Photosynth. Res. 25, 147–150. doi: 10.1007/BF00033156 24420345

[B40] von Caemmerer S. (2000). Biochemical models of leaf photosynthesis (Victoria, Australia: CSIRO Publishing).

[B41] von Caemmerer S. (2013). Steady-state models of photosynthesis. Plant Cell Environ. 36, 1617–1630. doi: 10.1111/pce.12098 23496792

[B42] Wang B. Zhuang Z. Zhang Z. Draye X. Shuang L.-S. Shehzad T.. (2017). Advanced backcross QTL analysis of fiber strength and fineness in a cross between *Gossypium hirsutum* and *G. mustelinum* . Front. Plant Sci. 8. doi: 10.3389/fpls.2017.01848 PMC5661169 29118778

[B43] Webb W. L. Newton M. Starr D. (1974). Carbon dioxide exchange of *Alnus rubra* . Oecologia 17, 281–291. doi: 10.1007/BF00345747 28308943

[B44] Wellburn A. R. (1994). The spectral determination of chlorophylls a and b, as well as total carotenoids, using various solvents with spectrophotometers of different resolution. J. Plant Physiol. 144, 307–313. doi: 10.1016/S0176-1617(11)81192-2

[B45] Xiao Y. Tholen D. Zhu X.-G. (2016). The influence of leaf anatomy on the internal light environment and photosynthetic electron transport rate: exploration with a new leaf ray tracing model. J. Exp. Bot. 67, 6021–6035. doi: 10.1093/jxb/erw359 27702991 PMC5100017

[B46] Yang X. L. Dong W. Liu L. H. Bi Y. H. Xu W. Y. Wang X. (2023). Uncovering the differential growth of *Microcystis aeruginosa* cultivated under nitrate and ammonium from a pathophysiological perspective. ACS ES&T Water 3, 1161–1171. doi: 10.1021/acsestwater.2c00624

[B47] Yang X.-L. Ma X.-F. Ye Z.-P. Yang L.-S. Shi J.-B. Wang X.. (2024). Simulating short-term light responses of photosynthesis and water use efficiency in sweet sorghum under varying temperature and CO_2_ conditions. Front. Plant Sci. 15. doi: 10.3389/fpls.2024.1291630 PMC11007071 38606074

[B48] Ye Z.-P. An T. Govindjee G. Robakowski P. Stirbet A. Yang X.-L.. (2024). Addressing the long-standing limitations of double exponential and non-rectangular hyperbolic models in quantifying light-response of electron transport rates in different photosynthetic organisms under various conditions. Front. Plant Sci. 15. doi: 10.3389/fpls.2024.1332875 PMC10929714 38476692

[B49] Ye Z. P. Robakowski P. Suggett D. J. (2013a). A mechanistic model for the light response of photosynthetic electron transport rate based on light harvesting properties of photosynthetic pigment molecules. Planta 237, 837–847. doi: 10.1007/s00425-012-1790-z 23138268

[B50] Ye Z. P. Suggett J. D. Robakowski P. Kang H. J. (2013b). A mechanistic model for the photosynthesis-light response based on the photosynthetic electron transport of photosystem II in C_3_ and C_4_ species. New Phytol. 199, 110–120. doi: 10.1111/nph.12242 23521402

[B51] Yin X. Busch F. A. Struik P. C. Sharkey T. D. (2021). Evolution of a biochemical model of steady-state photosynthesis. Plant Cell Environ. 44, 2811–2837. doi: 10.1111/pce.14070 33872407 PMC8453732

[B52] Yin X. Struik P. C. Romero P. Harbinson J. Vos J. (2009). Using combined measurements of gas exchange and chlorophyll fluorescence to estimate parameters of a biochemical C_3_ photosynthesis model: a critical appraisal and a new integrated approach applied to leaves in a wheat (*Triticum aestivum*) canopy. Plant Cell Environ. 32, 448–464. doi: 10.1111/j.1365-3040.2009.01934.x 19183300

